# Intranasal dexmedetomidine for procedural sedation in preschool children aged 2–6 years undergoing emergency gastric lavage for acute poisoning: a propensity score-matched single-center retrospective cohort study

**DOI:** 10.3389/fphar.2026.1921999

**Published:** 2026-09-21

**Authors:** Wenbin Gao, Yanke Yue, Wenjin Geng, Pei Zhao, Xiao Wang, Yanhua Feng

**Affiliations:** 1 Department of Emergency Surgery, Hebei Children’s Hospital, Hebei Provincial Clinical Research Center for Child Health and Disease, Shijiazhuang, China; 2 Department of Emergency, Hebei Children’s Hospital, Hebei Provincial Clinical Research Center for Child Health and Disease, Shijiazhuang, China

**Keywords:** agitation, dexmedetomidine hydrochloride, gastric lavage, medical resource utilization, pediatric emergency, procedural compliance

## Abstract

**Objective:**

This study aimed to evaluate the sedative efficacy and peri-procedural safety of preemptive intranasal dexmedetomidine in preschool children receiving emergency gastric lavage due to acute poisoning. Satisfactory procedural compliance rate was predefined as the primary efficacy outcome, and the total incidence of sedation-related adverse events was set as the primary safety outcome. Other behavioral scores, procedure time, vital signs and individual adverse reactions were regarded as secondary exploratory outcomes, and Bonferroni correction was used to correct multiple test bias for secondary indicators. We further quantified its benefits in enhancing procedural compliance, stabilizing peri-procedural vital signs, shortening rescue duration, and optimizing emergency staffing allocation—key practical challenges in overcrowded pediatric emergency departments.

**Methods:**

We conducted a retrospective cohort study of 200 children undergoing emergency gastric lavage for acute poisoning. After propensity score matching (1:1), 100 children received intranasal dexmedetomidine (2 μg/kg) 10 min before the procedure combined with routine comfort care (observation group), and 100 received comfort care and physical restraint only (control group). Procedural compliance, agitation, hemodynamic and respiratory parameters, procedural efficiency, and adverse events were compared between the two groups. Procedural compliance rate served as the predefined primary efficacy outcome, while total adverse event incidence was the primary safety outcome; all remaining indicators were secondary exploratory outcomes adjusted via Bonferroni correction.

**Results:**

Baseline characteristics were well balanced after matching. The observation group had a significantly higher effective compliance rate (93.0% vs. 60.0%) and first-attempt orogastric tube placement success rate (93.0% vs. 64.0%), and a lower agitation rate (7.0% vs. 40.0%) (all P < 0.001). Intraoperative heart rate, respiratory rate, and mean arterial pressure were significantly more stable and remained within normal physiological ranges in the observation group. This sedation protocol shortened lavage duration by 8.90 min, reduced orogastric tube placement attempts and staffing requirement (2.76–1.82 staff per case) (all P < 0.001), greatly improving rescue efficiency. No serious adverse events were observed in this selected cohort in either group, and all mild events resolved spontaneously.

**Conclusion:**

Preemptive intranasal dexmedetomidine may serve as a safe, non-invasive sedative alternative for emergency gastric lavage among selected young children with acute poisoning. In this cohort, it was associated with improved procedural compliance and more stable peri-procedural vital signs. This regimen was also associated with shortened procedural duration and optimized emergency staffing resources, which may be valuable in overcrowded pediatric emergency departments. Observations of rapid onset, favorable tolerability and low frequency of respiratory-related events in the present cohort warrant further investigation in larger pediatric emergency populations.

## 1 Introduction

Acute poisoning constitutes a life-threatening pediatric emergency and a top cause of unintentional childhood death, contributing to a large share of pediatric emergency department admissions globally (Di Sarno et al., 2025). Children aged 1–6 years represent a high-risk population for acute poisoning owing to immature physical and psychological development, limited cognitive function, poor self-protection and behavioral regulation, intense curiosity, and impaired risk recognition (Singer et al., 2024). Common toxic exposures in routine clinical practice include accidental drug overdose, household chemical ingestion, and consumption of toxic plants. In our regional clinical cohort, most poisoning incidents occurred at home under insufficient supervision by elderly caregivers, and accidental ingestion of adults’ oral antihypertensive and hypoglycemic tablets constituted the predominant toxic exposure. These solid chronic disease medications are rapidly absorbed in the digestive tract and carry high risks of severe hypotension or refractory hypoglycemia once ingested. Most guardians rushed to the emergency department immediately after discovering accidental ingestion, and timely hospital presentation provided a favorable therapeutic window for gastric decontamination.

Activated charcoal aqueous slurry is recommended for gastrointestinal decontamination in many poisoning guidelines, yet oral administration requires full voluntary compliance of children. For unsedated preschool children aged 2–6 years with intense fear and violent agitation, complete intake of the full therapeutic charcoal dose cannot be guaranteed. Forced feeding often triggers severe vomiting, accompanied by substantial risks of charcoal aspiration, airway obstruction and aspiration pneumonitis. For patients presenting promptly after ingestion with intact solid tablet residues retained in the stomach, sedation-assisted gastric lavage can directly and thoroughly clear undissolved toxic fragments, making it a more suitable standardized decontamination strategy for this specific population. Failure to timely and thoroughly eliminate residual gastrointestinal toxins may rapidly trigger multiple organ dysfunction syndrome, which can be life-threatening in severe cases. Gastric lavage was selectively performed for children retaining intact solid oral toxicants within 4 h post-ingestion per hospital protocols.

Gastric lavage is an invasive intervention that induces intense foreign-body sensation, nausea, discomfort, and pain stimulation. Acute toxic exposure frequently induces severe psychological distress in children; parental anxiety and panic further amplify pediatric fear, triggering violent resistance including inconsolable crying, limb thrashing, head torsion, and jaw clenching, which markedly impairs procedural compliance. Pediatric agitation complicates orogastric tube placement, necessitates repeated orogastric tube placement attempts, and elevates the risk of nasopharyngeal and esophageal mucosal injury, hemorrhage, and edema, as well as severe complications including laryngospasm and pulmonary aspiration (Runge et al., 2023). At the same time, it disturbs the operative field, prolongs the procedure time, reduces the decontamination efficiency of gastric lavage, and delays the optimal treatment time. Sustained struggling can lead to elevated heart rate and blood pressure, increased oxygen consumption, exacerbated stress response in poisoned children, and secondary physiological injury. Severe prolonged crying also raises intrathoracic pressure and causes agitation-related facial petechiae without organic capillary damage (Soares et al., 2020). Accordingly, under the premise of guaranteeing clinical safety, developing standardized sedative interventions to improve procedural compliance, shorten procedure duration, reduce complication rates, and optimize medical resource allocation has become an urgent unmet clinical need in pediatric emergency medicine.

At present, common sedatives for pediatric procedural sedation include chloral hydrate, midazolam, and propofol, all of which have obvious limitations in clinical application. Chloral hydrate is primarily administered via oral or rectal routes; it exhibits slow onset and inconsistent sedation depth, is prone to adverse effects including nausea, vomiting, and paradoxical agitation, and is unsuitable for emergency pediatric patients with a full stomach (Jang et al., 2025). As a benzodiazepine drug, midazolam has both sedative and anxiolytic effects, but it may induce respiratory depression, delayed recovery, and excitatory agitation, with limited safety in full-stomach, poisoned, or high-airway-risk children (Jang and Kim, 2024). Propofol has a strong and rapid sedative effect, but requires intravenous administration, causes significant respiratory and circulatory depression, and requires full monitoring by anesthesiologists, making it difficult to implement rapidly at the emergency bedside (Jang and Kim, 2024). Collectively, pediatric emergency environments urgently require a sedative agent characterized by rapid onset, titratable arousable sedation, minimal risk of respiratory depression, non-invasive delivery, and a favorable overall safety profile. Few studies have systematically evaluated intranasal dexmedetomidine for emergency resource optimization, which this study addresses.

Dexmedetomidine hydrochloride is a highly selective α_2_-adrenergic receptor agonist, which mainly acts on the central locus coeruleus. It suppresses central norepinephrine release to elicit arousable physiological sleep-like sedation, alongside anxiolytic, mild analgesic, and sympatholytic properties (Jiang et al., 2025). Its core advantage is the absence of significant respiratory depression, which preserves children’s spontaneous breathing and airway protective reflexes, making it highly suitable for poisoned, full-stomach, and non-intubated pediatric emergency patients (Gregory et al., 2025). Intranasal delivery is non-invasive, and convenient, with rapid mucosal absorption and high acceptability among pediatric patients. This route eliminates the need for prior intravenous cannulation, delivers mild, titratable sedation with fast effect, recovery, and exhibits no prominent long-term adverse effects, fully satisfying clinical demands for rapid sedation in pediatric emergency settings (Rocha et al., 2025). In recent years, dexmedetomidine has been gradually applied in procedural sedation scenarios such as pediatric imaging examination, traumatic suture, and puncture procedures, with proven sedative efficacy (Khan et al., 2024). However, systematic controlled studies on procedural compliance, vital signs, procedural efficiency, and medical resource utilization in high-stress, high-irritation, and time-consuming emergency procedures such as pediatric emergency gastric lavage are still scarce.

Based on the above clinical background, the present study enrolled children aged 2–6 years who underwent emergency gastric lavage for acute poisoning at our hospital between January 2025 and December 2025. We systematically evaluated the sedative efficacy, effects on vital signs, procedural efficiency, and medical resource utilization of preemptive intranasal dexmedetomidine hydrochloride. This study sought to generate high-quality evidence to support the development of a standardized, safe, and efficient procedural sedation protocol for emergency gastric lavage within pediatric emergency departments.

## 2 Materials and methods

### 2.1 Study design

This was a single-center retrospective cohort study performed in strict compliance with the STROBE reporting guidelines (Tufan and Akturk, 2021).This retrospective study was reviewed and formally approved by the Medical Research Ethics Committee of Hebei Children’s Hospital (Ethics Approval No. 202407-83) and performed in strict accordance with the Declaration of Helsinki and its subsequent revisions. Considering that this study only analyzed de-identified historical electronic medical records without additional clinical intervention for enrolled children, the ethics committee granted a formal waiver of the requirement for written informed consent from guardians. All patient-related information was fully anonymized before statistical analysis to protect personal privacy, and no extra risks were brought to the included subjects.

### 2.2 Study objects

#### 2.2.1 Inclusion criteria

(1) Aged 2–6 years, sex unlimited; (2) Diagnosed with acute poisoning, presented to the hospital within 4 h after poisoning, and met the indications for emergency gastric lavage; (3) Patients met ASA physical status class I–II, were hemodynamically stable, and presented without shock, severe dyspnea, or altered mental status; (4) Gastric lavage was performed by attending physicians with ≥3 years of experience in pediatric emergency medicine; (5) Complete core data including baseline data, vital signs, procedural records, sedation assessment, and adverse events.

#### 2.2.2 Exclusion criteria

(1) Corrosive poisoning (strong acid, strong alkali), high risk of gastrointestinal perforation or bleeding; (2) Complicated with severe craniomaxillofacial injury, airway malformation, or risk of airway obstruction; (3) Complicated with severe cardiac, hepatic, renal, or coagulation dysfunction, arrhythmia, cardiomyopathy, or congenital heart disease; (4) Neurodevelopmental delay, epilepsy, mental illness, or long-term use of sedatives; (5) Hypersensitivity to dexmedetomidine; (6) Use of other sedatives, analgesics, or anxiolytics within 24 h before admission; (7) Contraindications to intranasal administration such as nasal malformation, nasal trauma, epistaxis, or postoperative nasal surgery; (8) Severely incomplete clinical data precluding efficacy and safety evaluation.

### 2.3 Grouping and propensity score matching

Children were divided into the observation group (intranasal dexmedetomidine hydrochloride + routine comfort care 10 min before the procedure) and control group (only routine comfort, parental company, necessary restraint, no sedatives) according to whether they received intranasal dexmedetomidine hydrochloride sedation before procedure. 1:1 propensity score nearest-neighbor matching was used to control selection bias: a logistic regression model was constructed with sedation intervention as the dependent variable, age, sex, body weight, time from poisoning to hospital visit, type of poisoning substance, preoperative heart rate, respiratory rate, oxygen saturation, and mean arterial pressure as independent variables, with a caliper value of 0.2 and no replacement matching. After matching, balance was determined by a standardized mean difference (SMD) < 0.2. Finally, 100 cases in the observation group and 100 cases in the control group were included, with a total sample size of 200 cases. These covariates were selected *a priori* based on their potential to influence both sedation decision and procedural outcomes, as identified in previous observational studies.

Notably, several clinical factors that may interfere with sedation selection and procedural performance could not be incorporated into the propensity score logistic regression model due to the limitations of retrospective electronic medical records. These unmeasured potential confounders included real-time emergency department crowding level on admission, clinical severity grading of poisoning manifestations, and parental willingness to accept pre-procedural sedation for children, all of which lacked standardized quantitative recording in our original nursing and medical records and thus could not be balanced via propensity score matching. A total of 272 children diagnosed with acute poisoning and receiving emergency gastric lavage were initially screened according to pre-defined inclusion and exclusion criteria. Twenty-six patients were excluded for failing to meet the eligibility standards, leaving a pre-matching candidate cohort of 246 cases, consisting of 127 children in the intranasal dexmedetomidine observation group and 119 children in the control group receiving only physical restraint and comfort care.

We conducted 1:1 nearest-neighbor propensity score matching without replacement. To comply with standardized statistical reporting standards, the caliper width was defined as 0.2 standard deviations of the logit-transformed propensity score rather than a simple absolute value of 0.2. A fixed random seed was applied to guarantee the repeatability of matching results, and the average treatment effect on the treated (ATT) was set as the primary target estimand of this study. During matching, 46 participants were eliminated due to out-of-common-support propensity scores or absence of eligible matched counterparts within the caliper range. No matching covariate contained missing data exceeding 5%, which would not compromise matching stability.

We adopted a stricter standardized mean difference (SMD) threshold of <0.2 to judge covariate balance instead of merely relying on P values, as P values are inappropriate for evaluating post-matching balance. A sensitivity analysis integrating multivariable regression adjustment and inverse probability weighting (IPW) was performed to verify the robustness of primary research outcomes. After the above matching procedures, 100 matched pairs (200 patients in total) were finally enrolled for subsequent statistical analyses.

The 200 patients analyzed in all outcome tables only represent the 100 matched pairs obtained after propensity score matching, which were selected from the initial 272 screened children after exclusion and matching screening; unmatched individuals were not included in formal outcome analysis.

### 2.4 Intervention measures

The procedural duration of gastric lavage was defined as the time from the start of orogastric tube insertion to the completion of the final lavage fluid drainage. One placement attempt was defined as a single continuous insertion procedure; pulling the tube out completely and reinserting was counted as a new separate attempt. Correct intragastric tube position was confirmed by three methods: aspiration of gastric fluid, auscultation of air insufflation over the epigastrium, and observation of no respiratory distress during fluid injection. Uniform lavage fluid type, total volume and lavage cycles were applied to all enrolled children according to standardized emergency protocol; no differences in lavage parameters existed between the observation group and the control group. All gastric lavage procedures were performed by a fixed team of emergency pediatric physicians who treated patients in both groups without grouping preference. The number of medical staff was pre-specified before the procedure according to the emergency standard operating procedure, not adjusted based on children’s agitation performance. The physical restraint method and strength were standardized for all subjects. The data of procedure duration and staff number were recorded in real time during the procedure.

#### 2.4.1 Observation group

Intranasal dexmedetomidine hydrochloride spray (Aibeimei®) at a dose of 2 μg/kg was administrated equally into bilateral nasal cavities 10 min before planned gastric lavage, combined with standardized routine comfort care. As clearly stated in the official drug instructions and phase III clinical trial data of this preparation, this spray is only approved for preoperative sedation of children aged 2–6 years undergoing elective general anesthesia; its application for sedation during emergency gastric lavage for acute poisoning in the present study represents off-label use.

The phase III clinical trial results indicated that mild anxiolysis and early sedative effects can be detected 10 min after intranasal administration, while the median time to reach moderate sedation (Ramsay sedation score ≥ 3) was 20 min, with effective sedation maintained for 30–60 min. In this study, gastric lavage was not initiated mechanically at the 10-min time point. Instead, standardized bedside sedation assessment was performed 10 min after nasal spraying: only children who kept quiet with closed eyes and were easily aroused by mild stimulation proceeded to gastric lavage.

All clinical staff involved completed institutional training covering pediatric procedural sedation and airway rescue. A dedicated emergency physician monitored each child’s sedation, breathing and hemodynamics separately from the lavage operator. Continuous ECG and pulse oximetry were applied for all patients; end-tidal CO_2_ monitoring was added for high-airway-risk children. Rescue medications and airway equipment were kept available bedside at all times. Any child failing to reach satisfactory sedation after intranasal drug administration was excluded before matching to avoid grouping bias.

#### 2.4.2 Control group

No sedatives, analgesics, or anxiolytic medications were administered. Patients received only routine comfort support and verbal guidance with parental accompaniment; moderate physical and cranial restraints were implemented for severe agitation.

#### 2.4.3 Standardized procedure of gastric lavage

Both groups underwent the standardized Pediatric Emergency Gastric Lavage Procedure uniformly: an orogastric tube was selected according to age, 0.9% normal saline at 37 °C–38 °C was used for irrigation, a single dose of 10 mL/kg per cycle (maximum 200 mL per cycle), with repeated irrigation until the effluent was clear, odorless, and free of particulate matter. Electrocardiogram, heart rate, respiratory rate, oxygen saturation, and non-invasive blood pressure were monitored throughout the procedure.

### 2.5 Outcome measures

We prespecified primary efficacy outcome, primary safety outcome and multiple secondary outcomes before data analysis to avoid multiplicity bias caused by excessive statistical comparisons.

1. Primary efficacy outcome.

The rate of satisfactory procedural compliance during gastric lavage was defined as the primary efficacy indicator, which reflects the core clinical value of sedation for smooth completion of emergency gastric lavage. Procedural compliance was assessed by the Houpt overall behavioral rating scale. Scores of 4–6 were defined as effective procedural compliance, while scores of 1–3 were classified as agitation and poor compliance. The distribution of Houpt scores, effective compliance rate and agitation incidence with 95% confidence interval (CI) were recorded. The University of Michigan Sedation Scale (UMSS) only evaluates sedation depth and cannot objectively reflect behavioral interference during gastric lavage; therefore, the Houpt overall behavioral rating scale dedicated to assessing pediatric procedural compliance was adopted as the core evaluation basis for the primary outcome (Poonyarit et al., 2025). Although UMSS was considered during study planning, it was only used for auxiliary sedation-depth monitoring and was not applied to evaluate procedural compliance. The primary efficacy endpoint (satisfactory procedural compliance rate) was pre-specified before data analysis. Procedural compliance was derived from prospectively documented intraoperative behavioral narratives within electronic medical records, which were retrospectively categorized according to Houpt scale criteria during data analysis; no new *post hoc* patient-level data were generated. All compliance assessments relied on bedside behavioral descriptions prospectively recorded by trained emergency clinicians, rather than scores assigned after observing study outcomes.

2. Primary safety outcome.

The total incidence of all sedation-related adverse events was set as the primary safety outcome to evaluate the overall safety profile of preemptive intranasal dexmedetomidine. Sedation-related adverse events were predefined as any untoward clinical event temporally associated with study-related sedation, including respiratory depression, severe bradycardia, laryngospasm, pulmonary aspiration, gastrointestinal perforation, nasal irritation, nausea, vomiting, and agitation-related pressure petechiae (pinpoint hemorrhages on the head and face). All sedation-related adverse events and real-time monitoring data including heart rate, respiratory rate, SpO_2_ and ETCO_2_ (for high-risk patients) were fully recorded during the whole sedation and lavage period.

3. Secondary outcomes.

The following indicators were collected as secondary exploratory outcomes: procedural duration, first-attempt orogastric tube placement success rate, total orogastric tube placement attempts, medical manpower input per patient; peri-procedural vital signs including heart rate (HR), respiratory rate (RR), oxygen saturation (SpO_2_), and mean arterial pressure (MAP) (baseline values, mean values at 5 min, 10 min, and the end of the procedure during lavage).

For multiple statistical comparisons among these secondary outcomes, the Bonferroni correction was adopted to control the type I error and reduce the probability of false-positive results caused by repeated testing.

In addition, baseline demographic and clinical data were collected for covariate balance assessment, including age, sex, weight, time from poisoning to hospital visit, and type of poisoning substance.

### 2.6 Statistical analysis

Data were analyzed using SPSS 30.0 software. All statistical tests were two-tailed, and a P-value <0.05 was considered statistically significant. One-to-one nearest-neighbor propensity score matching was performed to balance baseline confounding factors. Covariate balance was evaluated only by standardized mean difference (SMD), and SMD<0.2 was regarded as adequate balance. P-values were not used to assess baseline balance. Normally distributed continuous variables were expressed as mean ± standard deviation and analyzed via paired t-test. Non-normal continuous data were presented as median (interquartile range) and compared with Wilcoxon signed-rank test. Categorical data were analyzed using McNemar test. Ordinal behavioral scores were assessed by Wilcoxon signed-rank test. A two-sided test was used, and P < 0.05 was considered statistically significant. Notably, this study adopted propensity score matched paired samples; statistical tests suited paired data (paired t-test, Wilcoxon signed-rank test, McNemar test) were applied for intergroup comparison rather than independent sample tests to avoid statistical bias caused by matched pairing relationships. Multiple comparisons of secondary outcome indicators were adjusted by the Bonferroni method to avoid inflated type I error due to repeated statistical testing. In addition, baseline demographic and clinical data were collected for covariate balance assessment, including age, sex, weight, time from poisoning to hospital visit, and type of poisoning substance. A linear mixed-effects repeated-measures model was used to analyze the dynamic changing trend of heart rate, respiratory rate and MAP over time. The change value from baseline, within-group standard deviation and coefficient of variation were calculated to assess the fluctuation and hemodynamic stability of vital signs, rather than only comparing the absolute mean values between groups.

Normality of continuous variables was evaluated using the Shapiro-Wilk test combined with visual Q-Q plot inspection to select appropriate statistical approaches. All incomplete medical records with key outcome variables missing were excluded from the analysis via complete-case analysis; multiple imputation was not performed due to low missing proportion (<5%). This limitation is acknowledged in the Discussion section. Sample size was not pre-calculated via power analysis due to retrospective study design; all consecutive eligible patients within the study period were enrolled for 1:1 propensity score matching.

## 3 Results

### 3.1 Comparison of baseline data

A total of 200 children were included in this study, with 100 cases in each group. After propensity score matching, the baseline data of the two groups achieved good balance and comparability according to the preset SMD standard (Table 1).

**TABLE 1:** Baseline characteristics of the two groups after propensity score matching. Covariate balance was evaluated by standardized mean difference (SMD).

Index	Observation group (n = 100)	Control group (n = 100)	SMD
Age (years)	4.08 ± 0.94	4.03 ± 0.98	0.052
Sex (male/female)	56/44	53/47	0.060
Weight (kg)	15.47 ± 2.08	15.25 ± 2.15	0.104
Time from poisoning to hospital (min)	67.8 ± 20.9	69.5 ± 22.8	0.078
Preoperative HR (bpm)	112.9 ± 9.8	112.3 ± 9.5	0.062
Preoperative RR (/min)	24.4 ± 3.0	24.1 ± 3.2	0.097
Preoperative SpO_2_ (%)	98.4 ± 1.0	98.2 ± 1.2	0.179
Preoperative MAP (mmHg)	82.3 ± 6.1	81.7 ± 6.4	0.096

Further stratified epidemiological analysis of baseline data demonstrated that drug poisoning accounted for the largest proportion of all toxic exposures, mainly accidental ingestion of adult antihypertensive and hypoglycemic drugs. The vast majority of children received prompt medical consultation after poisoning, meeting the clinical indications for emergency gastric lavage to clear unabsorbed solid gastric toxins. Although most covariates had a low SMD value after matching, the preoperative SpO_2_ had an SMD of 0.179, which was still within the acceptable balance threshold of SMD <0.2. No statistically significant intergroup differences were observed in all baseline indicators (all P ≥ 0.05).

### 3.2 Comparison of procedural compliance and agitation incidence (Houpt overall behavioral scale)

The effective compliance rate (Houpt overall score ≥4) in the observation group was 93.00%, significantly higher than 60.00% in the control group. The agitation incidence (Houpt overall score ≤3) in the observation group was 7.00%, significantly lower than 40.00% in the control group. The difference between groups was statistically significant (P < 0.001). No child achieved Houpt score 1 (severe agitation leading to repeated procedural interruption and difficulty completing lavage) in the observation group, while 14.00% of children in the control group showed severe agitation corresponding to Houpt score 2 (Table 2). Group difference in effective compliance rate = 33.0% (95%CI 21.2%–44.8%, P < 0.001).

**TABLE 2:** Comparison of procedural compliance and agitation between groups (Houpt overall behavioral scale).

Indicators	Observation (n = 100)	Control (n = 100)	P Value
Effective procedural compliance (Houpt overall behavioral score ≥4), n(%)	93.0%	60.0%	<0.001
Agitation (Houpt score≤3), n(%)	7.0%	40.0%	<0.001
Median Houpt score [ P25, P75]	5 [4,6]	3 [2,4]	<0.001

Effective compliance was defined as Houpt overall score ≥4, while agitation/poor compliance was defined as Houpt overall score ≤3. Houpt score 1: Severe agitation, gastric lavage cannot be completed smoothly; Houpt score 2: Persistent screaming and limb twisting, the procedure is frequently interrupted; Houpt score 3: Intermittent crying and mild struggling, lavage can be finished after intervention; Houpt score 4: Slight transient discomfort without obvious interference with procedure; Houpt score 5: Only brief crying during orogastric tube placement, stable state for the rest procedure; Houpt score 6: No crying or limb struggling throughout the whole gastric lavage.

### 3.3 Comparison of medical resource utilization indexes

The observation group was significantly superior to the control group in procedural duration, first-attempt orogastric tube placement success rate, orogastric tube placement times, and medical manpower input. The mean gastric lavage duration in the observation group was 22.55 ± 3.41 min, while that in the control group was 31.45 ± 4.73 min, with an average reduction of 8.90 min (P < 0.001) (Table 3). Mean difference of lavage duration = −8.90 min (95%CI −10.6∼−7.2, P < 0.001).

**TABLE 3:** Comparison of procedural efficiency and medical resource utilization.

Indicators	Observation group (n = 100)	Control group (n = 100)	P Value
One-time orogastric tube placement success rate, n (%)	93.0%	64.0%	<0.001
Number of tube placements	1.07 ± 0.26	1.70 ± 0.96	<0.001
Total gastric lavage procedure time (min), mean ± SD	22.55 ± 3.41	31.45 ± 4.73	<0.001
Medical staffing per procedure (staff/case), mean ± SD	1.82 ± 0.42	2.76 ± 0.68	<0.001

P values were calculated using paired t-test (procedure time and staffing), McNemar test (success rate), and Wilcoxon signed-rank test (number of attempts).

### 3.4 Comparison of vital signs

Intraoperative heart rate and SpO_2_ were significantly lower in the observation group (both P < 0.001), while there was no significant intergroup difference in MAP (P = 0.193). SpO_2_ of all patients remained above 95% throughout the gastric lavage procedure (Table 4).

**TABLE 4:** Comparison of vital signs.

Vital signs	Observation (n = 100)	Control (n = 100)	P Value
HR (bpm)	91.65 ± 9.11	117.25 ± 14.8	<0.001
RR (/min)	19.38 ± 2.13	29.32 ± 4.48	<0.001
SpO_2_ (%)	97.66 ± 1.18	97.86 ± 1.06	<0.001
MAP (mmHg)	76.69 ± 6.25	87.02 ± 8.45	0.193

### 3.5 Comparison of the changes in vital signs from baseline to intraoperative period

We further calculated the difference values of heart rate, respiratory rate and mean arterial pressure between the baseline level and the average intraoperative level of the two groups to analyze the fluctuation range of vital signs during gastric lavage. The results of vital sign changes are shown in Table 5.

**TABLE 5:** Changes in vital signs between baseline and procedure.

Changes from baseline (intraoperative-baseline)	Observation group (mean ± SD)	Control group (mean ± SD)	P Value
ΔHR (times/min)	−21.50 ± 13.98	5.01 ± 18.83	<0.001
ΔRR (times/min)	−3.55 ± 1.11	3.90 ± 3.25	<0.001
ΔMAP (mmHg)	−5.88 ± 1.30	5.54 ± 4.44	<0.001

### 3.6 Comparison of safety

No serious adverse events such as respiratory depression, severe bradycardia, laryngospasm, aspiration, or gastrointestinal perforation occurred in either group. The incidence of agitation-related pressure petechiae was 9.00% in the control group and 0% in the observation group (P = 0.002). The total incidence of adverse events was 3.00% in the observation group and 11.00% in the control group (P = 0.045) (Table 6).

**TABLE 6:** Comparison of adverse events.

Adverse event	Observation	Control	χ^2^	P Value
Transient bradycardia	2 (2.00)	6 (6.00)	1.042	0.307
Nasal irritation	1 (1.00)	0 (0.00)	1.005	0.316
Nausea	0 (0.00)	3 (3.00)	3.046	0.081
Vomiting	0 (0.00)	2 (2.00)	2.020	0.155
Agitation-related petechiae	0 (0.00)	9 (9.00)	9.424	0.002
Total patients with≥1 event, n(%)	3 (3.00)	11 (11.00)	4.031	0.045

Data are presented as number of patients with at least one adverse event (%). In the control group, 11 patients experienced a total of 21 events. No serious adverse events (e.g., respiratory depression, severe bradycardia, laryngospasm, aspiration, or gastrointestinal perforation) occurred in either group. P values were calculated using McNemar test.

## 4 Discussion

This single-center retrospective cohort study systematically evaluated the sedative efficacy, procedural efficiency, vital sign stability, and medical resource utilization value of intranasal dexmedetomidine hydrochloride for pediatric emergency gastric lavage. Our results suggested that the observation group was associated with markedly improved procedural compliance and first-pass orogastric tube placement success, alongside reduced agitation rates, shorter procedural duration, and lower staffing demand. Intraoperative cardiovascular and respiratory parameters remained more stable, and no severe adverse events were recorded. Collectively, findings from this matched cohort suggest that intranasal dexmedetomidine may be a well-tolerated sedative option for pediatric emergency gastric lavage, and it was associated with improved procedural compliance and optimized emergency resource allocation in our study population. From an epidemiological perspective, drug poisoning dominated all toxic exposures in this cohort, largely attributable to inadequate supervision by elderly family members and easy access to adults’ oral antihypertensive and hypoglycemic tablets. Almost all patients sought emergency medical care promptly after ingestion, which created optimal conditions for gastric lavage to clear intact solid pharmaceutical residues before extensive gastrointestinal absorption.

Gastric lavage was set as the standardized gastrointestinal decontamination strategy in this study instead of routine oral activated charcoal. The relevant safety and efficacy reasons for this choice have been described in the study design section, which mainly takes the high aspiration risk of oral charcoal in resistant children and the characteristics of solid tablet poisons in our subjects into consideration. Activated charcoal was only used as an optional supplementary measure for individual patients after gastric lavage.

Poor procedural compliance during pediatric emergency gastric lavage is a common clinical problem in pediatric emergency medicine. Young children have limited cognitive and behavioral control abilities and strong fear of invasive procedures. Without sedation intervention, they are prone to violent crying and limb struggling, which increases the difficulty of orogastric tube placement and the risk of repeated orogastric tube placement, induces mucosal injury and aspiration, prolongs procedural duration, and increases medical burden (Çelikol et al., 2019). In our cohort, the control group received only routine comfort care and physical restraint without sedative intervention, with an effective compliance rate of only 60.00% and an agitation incidence of 40.00%, which conforms to routine clinical practice in pediatric emergency poisoning rescue. In the control group, 9.00% of children developed pinpoint hemorrhages on the head, neck, and upper chest after gastric lavage. These petechiae were previously hypothesized to relate to elevated intrathoracic pressure induced by severe crying and physical resistance; however, definite causal inference cannot be drawn from this retrospective dataset, and no evidence of permanent capillary injury or organic iatrogenic damage was observed in these cases (Ibraheim and Gavvala, 2021). Such petechiae not only indicate acute stress but may also signify elevated intracranial pressure or capillary fragility, which could be particularly hazardous in poisoned children with potential coagulopathy. After preoperative intranasal administration of dexmedetomidine hydrochloride in the observation group, the effective compliance rate increased to 93.00%, the agitation incidence decreased to 7.00%, and no severe agitation or agitation-related pressure petechiae occurred, suggesting a potential sedation-related intervention effect with this cohort. As a highly selective α_2_-adrenergic receptor agonist, dexmedetomidine acts on the central locus coeruleus, inhibits the release of norepinephrine, produces arousable physiological sedation, and has both anxiolytic and mild analgesic effects, which can effectively relieve children’s procedural fear and physical discomfort and reduce agitation and resistance (Ciavola et al., 2024).

Intranasal administration is an important advantage of dexmedetomidine suitable for pediatric emergency. This route is non-invasive, convenient, rapidly absorbed, no need for intravenous access, avoids secondary stress caused by puncture, has high acceptance by children, and is suitable for rapid bedside implementation in emergency (Gao et al., 2026). In this study, intranasal administration was completed 10 min before gastric lavage, and the onset speed and sedation depth could meet the needs of short-term emergency procedures. This study further confirmed that dexmedetomidine has an extremely low risk of respiratory depression. SpO_2_ during procedure in both groups was maintained above 95%, and no respiratory depression adverse events occurred. It has outstanding safety advantages for high-risk children such as full stomach, acute poisoning, and non-orogastric tube placement, and higher clinical safety compared with traditional sedatives such as midazolam and propofol (Freriksen et al., 2022). Furthermore, intranasal administration circumvents the need for intravenous access, which is often challenging in distressed young children and consumes additional nursing time—a critical factor in emergency triage. Intranasal dexmedetomidine can be used safely after the evaluation of emergency physicians, without the presence of anesthesiologists, which greatly optimizes the emergency treatment process and shortens the time from admission to gastric lavage (Bau et al., 2023).

Stable vital signs are the core of safety control in emergency sedation. Gastric lavage stimulation can activate the sympathetic nervous system, leading to abnormal elevation of heart rate, respiratory rate, and blood pressure, exacerbating systemic stress injury (Santos et al., 2021). The lower intraoperative HR, RR and MAP in the intranasal dexmedetomidine group are consistent with the inherent sympatholytic and mild hypotensive pharmacological effects of dexmedetomidine itself, which cannot be simply equated to hemodynamic stability. Further repeated-measures analysis showed that the observation group had smaller within-group variation of vital signs, slighter fluctuation range from baseline values, and fewer cases crossing age-adjusted clinical threshold values during the whole procedure. These findings suggest that the intervention group was associated with more stable hemodynamic performance during pediatric gastric lavage. This protective effect is particularly important for poisoned children, which can reduce heart rate acceleration, oxygen consumption increase, and circulatory fluctuation caused by agitation, reduce the risk of complications (Zheng et al., 2024), and ensure the safe implementation of gastric lavage.

Optimizing medical resource utilization and improving treatment efficiency are the core innovations of this study. Pediatric emergency resources are scarce, and procedural efficiency directly affects the overall treatment procedure and bed turnover (Gross et al., 2023). In the observation group, the average gastric lavage duration was 22.55 ± 3.41 min versus 31.45 ± 4.73 min in the control group, with an average reduction of 8.90 min. The one-time orogastric tube placement success rate rose from 64.00% in the control group to 93.00%. Shortened procedural duration can accelerate bed turnover and save consumables; improved first-attempt orogastric tube placement success rate can reduce airway and digestive tract injury and reduce subsequent diagnosis and treatment costs; reduced manpower consumption can effectively alleviate the workload of emergency medical staff (Ribeiro et al., 2025).

From the perspective of emergency hospital procedure management, this sedation protocol shortened the average lavage time by 8.90 min and reduced medical staff input per case from 2.76 to 1.82. Translated into clinical practice, shortened single-case treatment time accelerates emergency bed turnover and improves the overall rescue capacity of the ED, especially during peak poisoning admission periods. The reduction of one medical worker per procedure significantly alleviates the workload of emergency nurses and physicians, effectively relieving the staff shortage pressure in grassroots children’s hospitals. This economic and procedural benefit is an important innovation of this study, which is rarely reported in previous pediatric procedural sedation research.

Safety analysis showed that only transient mild bradycardia and slight nasal irritation occurred in the observation group, which could resolve spontaneously; nausea and vomiting in the control group were mostly caused by mechanical stimulation of gastric lavage. The overall incidence of adverse reactions was low in both groups, suggesting that intranasal dexmedetomidine is well tolerated in children aged 2–6 years, with no obvious respiratory or circulatory depression, and suitable for routine application in pediatric emergency. Agitation-related pressure petechiae, which was common in the control group, did not occur in the observation group, further indicating that sedation intervention can avoid microvascular injury induced by severe crying.

This study has certain limitations. First, it is a single-center retrospective study, and although propensity score matching was adopted to balance measurable baseline covariates, residual selection bias originating from unquantifiable clinical confounders cannot be completely eliminated. Several critical influencing factors mentioned in clinical practice were not available for matching and subgroup analysis in this retrospective cohort:

1. Real-time ED crowding could influence triage waiting time and children’s agitation, yet no standardized crowding grading was documented in medical records for covariate adjustment.
2. We enrolled only ASA I–II stable patients without stratifying by poisoning severity, which may mask subtle outcome differences across toxic severity subgroups.
3. Parental attitudes toward sedation were not routinely recorded in retrospective charts and could not be balanced via propensity matching.

Second, we did not compare intranasal dexmedetomidine with other sedative agents, as traditional sedatives require anesthesiologist supervision and are not routinely used in our emergency department for this indication. Third, the exact ingested dose of toxicants was not accurately recorded due to the retrospective nature of medical records. Fourth, long-term neurodevelopmental follow-up data were not collected for enrolled children. Multi-center prospective cohort research with standardized recording of ED crowding, poisoning severity and parental sedation acceptance is needed in future to further stratify and verify the efficacy of intranasal dexmedetomidine across different clinical scenarios.

## 5 Conclusion

In this single-center retrospective propensity-score-matched study, we enrolled children aged 2–6 years who underwent emergency gastric lavage. Intranasal dexmedetomidine sedation achieved acceptable Houpt behavioral scores, accompanied by mild fluctuations in respiratory function and hemodynamic indicators within age-appropriate physiological ranges in this selected cohort. The regimen may serve as a feasible alternative sedation option for pediatric emergency gastric lavage.

This study has obvious limitations: it is a retrospective single-center investigation with a moderate sample size of 100 matched pairs, no positive sedative control group, and strict inclusion and exclusion criteria that excluded critically ill children. Thus, the results cannot be widely extrapolated, and we cannot draw conclusions regarding its superiority over midazolam, propofol, ketamine and other common sedation schemes. Further well-designed prospective, multicenter controlled trials are required to verify its clinical efficacy and safety in broader pediatric emergency populations.

## Data Availability

The original contributions presented in the study are included in the article/supplementary material, further inquiries can be directed to the corresponding author.
